# Droplet Breakup in Expansion-contraction Microchannels

**DOI:** 10.1038/srep21527

**Published:** 2016-02-22

**Authors:** Pingan Zhu, Tiantian Kong, Leyan Lei, Xiaowei Tian, Zhanxiao Kang, Liqiu Wang

**Affiliations:** 1 Department of Mechanical Engineering, the University of Hong Kong, Hong Kong; 2 HKU-Zhejiang Institute of Research and Innovation (HKU-ZIRI), 311300, Hangzhou, Zhejiang, China

## Abstract

We investigate the influences of expansion-contraction microchannels on droplet breakup in capillary microfluidic devices. With variations in channel dimension, local shear stresses at the injection nozzle and focusing orifice vary, significantly impacting flow behavior including droplet breakup locations and breakup modes. We observe transition of droplet breakup location from focusing orifice to injection nozzle, and three distinct types of recently-reported tip-multi-breaking modes. By balancing local shear stresses and interfacial tension effects, we determine the critical condition for breakup location transition, and characterize the tip-multi-breaking mode quantitatively. In addition, we identify the mechanism responsible for the periodic oscillation of inner fluid tip in tip-multi-breaking mode. Our results offer fundamental understanding of two-phase flow behaviors in expansion-contraction microstructures, and would benefit droplet generation, manipulation and design of microfluidic devices.

In microfluidic channels, droplets are generated by injecting a liquid phase into another immiscible liquid. Drops break off from an orifice when shearing force and surface tension are balanced^1^. Assuming constant shearing force from outer phase, droplets with uniform sizes are generated one by one in a dripping manner. Attributed to the precise control of flow, the monodisperse, and size-controlled droplets generated by droplet microfluidics are extensively used for wide applications, ranging from foods^2^, pharmaceuticals^3^,^4^, cosmetics^5^ to materials synthesis^6^,^7^,^8^,^9^. These droplets can function as micro-reactors for chemical reactions^10^,^11^ and biological assays^12^,^13^,^14^ such as single-molecule polymerase chain reaction (PCR)^15^, or as carriers for active ingredients such as drugs^16^,^17^, proteins^18^ and cells^19^. Typically, as micro-reactors or carriers, droplets are merged in diverging channels to initiate chemical reactions^20^,^21^, or squeezed through narrow channels to probe the mechanical property of encapsulated protein networks^18^,^22^ and microcapsules^23^,^24^, or split into several daughter droplets^25^,^26^ for different biological assays^12^,^13^. Thus, channels with complex geometry are normally designed to facilitate manipulation of droplets, including mixing, splitting, diluting and fission^20^,^21^,^25^,^26^,^27^. Controlling the dynamical behaviors of individual droplets in complex channels is thus crucial for droplet-based applications^11^,^12^,^13^,^14^,^15^,^16^,^17^,^18^,^19^,^20^,^21^.

The geometry of microchannels, like expansions and contractions where flow velocity changes with the varying channel dimension, affects droplet behavior significantly. For example, the increase in flow velocity due to channel contraction increases the shearing force, leading to early breakup of the droplet from inner phase^24^. An inappropriate design in channel geometry can cause undesired droplet behaviors. For instance, when droplets are used as drug carriers in small vessels, these droplets should not split until arriving at the targeted site^28^. Moreover, as a large droplet is squeezed through a narrow channel, it may break into multiple daughter droplets in an uncontrolled manner^29^,^30^. This uncontrolled breakup of primary droplet significantly jeopardizes the uniformity of final droplets. Consequently, it is highly desired to systematically investigate and quantify the conditions of droplet breakup in complex channels, especially where expansions and contractions in channel dimensions are involved.

When injected into an immiscible outer fluid, the inner liquid can break up in different modes, including geometry-controlled^31^, dripping^7^,^32^,^33^, jetting^7^, tipstreaming^34^,^35^,^36^ and tip-multi-breaking^37^. The variation in channel geometry results in the transition between breakup modes, and thus changes the size and size distribution of final droplets. For instance, the increase of droplet size and a transition from dripping to geometry-controlled mode are observed, when the distance between two capillaries in flow-focusing capillary devices is increased^38^. Although quantified relationship between channel geometry and the size of final droplets is well studied for geometry-controlled, dripping, jetting and tipstreaming modes^7^,^36^,^39^, it is yet to be established for the recently reported tip-multi-breaking mode^37^, by which droplets are generated sequence by sequence with non-uniform size.

We investigate systematically the features of droplet breakup in capillary microfluidic devices with expansion-contraction configurations (Fig. 1a). The influences of varying channel geometry on flow behaviors are studied at the injection nozzle with diverging flow and at the focusing orifice with converging flow. Using local capillary numbers, we characterize the influences by conditions of droplet breakup, such as the location shift of droplet breakup and breakup mode transition. At the injection nozzle, we demonstrate that the local capillary number successfully predicts the condition for breakup location transition. At the focusing orifice, we highlight the transition of droplet breakup modes, and quantify both droplet size and number of droplets in the tip-multi-breaking mode^37^ (Fig. 1b). We also examine the mechanism responsible for evolution of inner liquid tip and the oscillation period of the tip. Our understanding of droplet breakup behavior influenced by the channel geometry, offers valuable guidelines for designing microchannels to generate and manipulate droplets in a precisely controlled manner.

## Experiments

**Figure 1:**
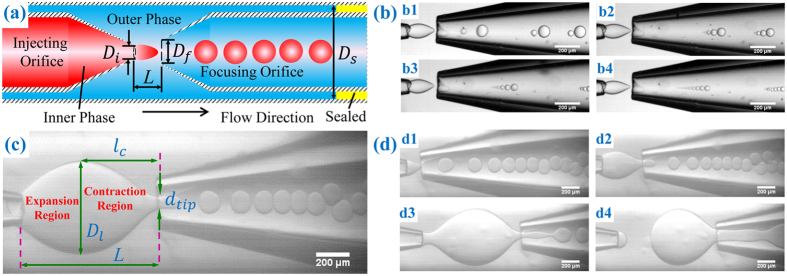
Device used and the influences of channel dimension on flow behaviors. (**a**) Schematic of the microcapillary device (not to scale). 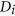, outer diameter of the injection nozzle; 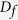, inner diameter of the focusing orifice; *L*, distance between two orifices; and 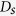, inner dimension of the square capillary. Both inner and outer fluids flow from left to right, and the gutter between the cylindrical and square capillary at right hand-side is sealed during operation (yellow region) to enhance flow focusing, but open at rest, so as to flush out wastes. (**b**) Droplets produced sequence by sequence in tip-multi-breaking mode with descending size distribution. (b1) 2-droplet sequence, (b2) 4-droplet sequence, (b3) 6-droplet sequence and (b4) 8-droplet sequence. (**c**) Geometrical parameters characterizing the expansion-contraction microchannel. 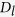, maximum diameter of the liquid tip; 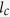, contraction distance; 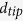, diameter of inner liquid tip at the focusing orifice. 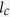 and 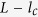 represent the length of contraction and expansion region, respectively. (**d**) Variation of inner fluid tip shape with enlarging orifice distance *L*. 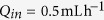, 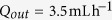. 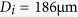, 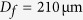. (d1) Cone-shape tip with 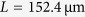, (d2) spindle-shape tip with 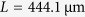, and (d3,d4) droplet breakup at injection nozzle with 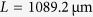. Time interval between (d3,d4) is 50 ms. Scale bars, 200 μm.

Capillary microfluidic devices were used to study the hydrodynamic behaviors of two-phase flows in microchannel with an expansion-contraction structure. The capillary microfluidic device was fabricated by aligning two tapered cylindrical glass capillaries inside a square capillary (inner dimension 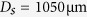), as shown in Fig. 1a. We varied three geometrical parameters 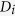, 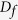 and *L* systematically, which were outer diameter of the injection nozzle, inner diameter of the focusing orifice, and distance between the two orifices, respectively. We summarized the combinations of geometrical parameters in Table 1. Three dynamic dimensions (Fig. 1c), the maximum diameter of inner liquid tip 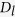, inner tip diameter at the focusing orifice 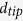 and the axial distance between the maximum inner tip and focusing orifice 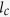, depend on both geometrical parameters 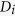, 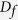 and *L* and the dynamic flow process (inner and outer flow rates *Q*_*in*_ and *Q*_*out*_, respectively). The length of contraction, where flow converges, is represented by 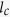; while that of the expansion, where flow diverges, is represented by 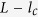 (Fig. 1c).

Both inner and outer fluids were injected into the microcapillary device from left to right, Fig. 1a. The flow rates for inner (*Q*_*in*_) and outer (*Q*_*out*_) phases were controlled by syringe pumps (Longer Pump). *Q*_*in*_ was experimentally confirmed to be constant with various orifice distance *L* (see “confirming constant inner flow rate” in supplementary information). The gutter between the cylindrical and square capillary at right hand side was sealed (yellow region in Fig. 1a) during operation, so as to enhance the flow contraction at the focusing orifice. The flow is visualized, monitored and recorded (images and videos) by a high-speed digital camera (MotionPro® X4, IDT, Taiwan, and Phantom M110) equipped with an inverted microscope (XD101, Nanjing Jiangnan Novel Optics Co. Ltd, and Nikon TS100). Captured images and videos were analyzed by ImageJ.

The fluid employed in experiments was water-in-oil two-phase flow, where the outer phase was silicone oil with fixed viscosity *η*_*out*_, and inner phase were various glycerol-water mixtures with different values of viscosity *η*_*in*_ (see supplementary Table S1). The mixture of 70 wt.% glycerol and 30 wt.% distilled water was used as inner phase fluid for most of the experiments, except that of determining the condition for the transition of droplet breakup location as a function of viscosity ratio *ξ* (defined as *ξ* = *η*_*in*_/*η*_*out*_). The viscosity was measured by a viscometer (microVISC^TM^, RheoSense, Inc.). *η*_*out*_ = 492.9 ± 6.9 mPa s for silicone oil, and *η*_*in*_ = 19.07 ± 0.12 mPa s for 70 wt.% glycerol (*ξ* = 0.039). The interfacial tension was measured by a ring tensiometer (Surface Tensiometer 20, Cole-Parmer) to be γ = 30.07 mN m^−1^ without any surfactants.

## Results and Discussion

### Influences of channel dimension on flow behaviors

The influence of channel geometry is manifested in the two local shear stresses at the injection nozzle and focusing orifice, respectively. Qualitatively, small value of *L* renders shear stress strong at the focusing orifice, but weak at the injection nozzle. When fluid flows in a channel with short distance *L*, the sharp contrast in local stresses leads to a cone-shape tip (Fig. 1d1). Droplets are generated at the focusing orifice where channel converges. With increasing *L*, the decreased difference in local stresses results in a spindle-shape tip (Fig. 1d2). However, if *L* increases further, the inner fluid tip is likely to break up at the injection nozzle where channel diverges (Fig. 1d3,d4). Therefore, the breakup location shifts from the focusing orifice to injection nozzle. Meanwhile, the detached drop from the injection nozzle would break up again into multiple daughter droplets with non-uniform size as it is squeezed into the focusing orifice (Fig. 1d3,d4, see supplementary movie S1). To produce uniform droplets, breakup at injection nozzle should thus be avoided.

**Figure 2:**
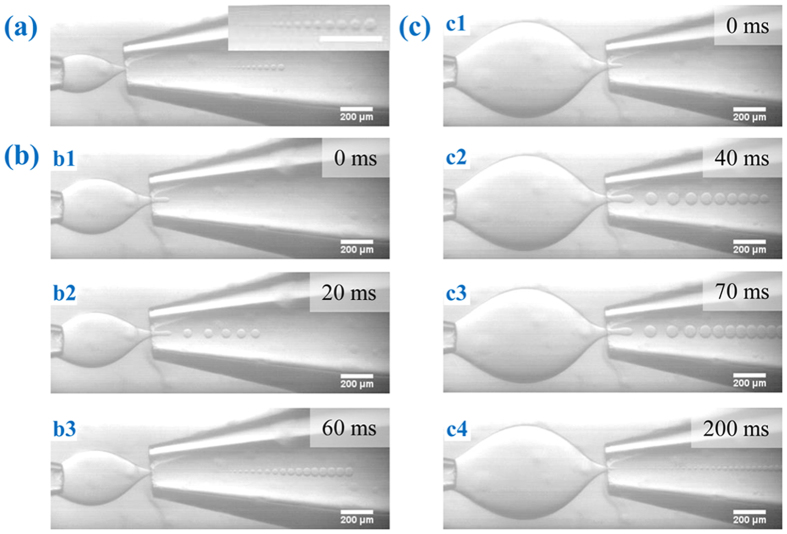
Effects of channel dimension on size distribution in tip-multi-breaking mode. All images are obtained with 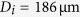, 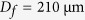, 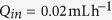 and 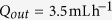. (**a**) Droplet sequence with descending sizes in one droplet train. 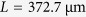. Inset: magnification of the droplet sequence. (**b**) Droplet size firstly keeps constant for dozens of milliseconds, and then deceases with time (“constant-decreasing”). 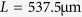. (**c**) Droplet size increases first, then keeps constant for a while, and finally decreases with time (“increasing-constant-decreasing”). 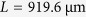. See supplementary movie S2 for details. Scale bars, 200 μm.

The variation in local shear stresses influences droplet breakup mode as well as droplet size distribution. As local shear stresses vary, the capillary number *Ca*, representing the ratio of shear stresses to surface tension, also changes. For instance, decrease of local capillary number at the focusing orifice by enlarging *L* leads to a transition from dipping to geometry-controlled mode, as observed by Benson *et al.*^38^ and in the present work. Interestingly, we also found the variation of tip-multi-breaking mode with *L* (Fig. 2, see supplementary movie S2). Tip-multi-breaking mode was previously reported with descending size distribution in one droplet train^37^ (Figs 1b and 2a). Here we find that the size distribution in one drop sequence can either keep constant for a while and then decrease (“constant-decreasing”, Fig. 2b), or increase first, then keep constant and finally decrease (“increasing-constant-decreasing”, Fig. 2c), depending on the orifice distance *L*.

**Figure 3:**
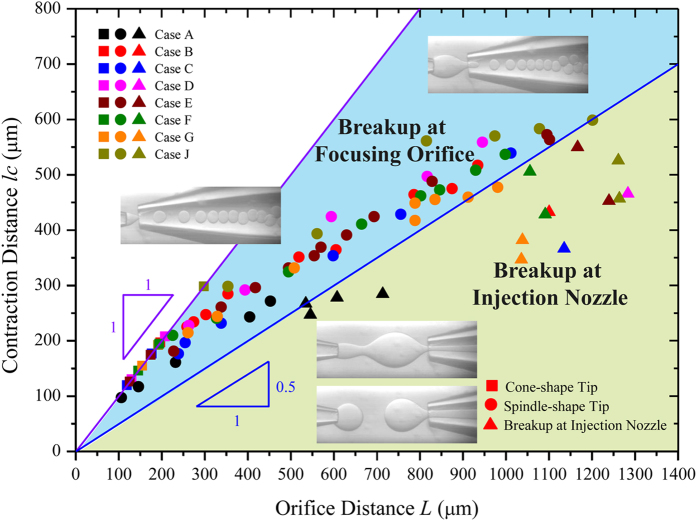
Phase diagram showing flow behaviors influenced by channel dimension. 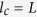
 represents cone-shape tip, while the region of 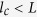 indicates the spindle-shape tip. 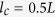 marks the transition of droplet breakup location from focusing orifice to injection nozzle. Different symbol colors represent different cases (Table 1). Different symbol shapes distinguish flow behaviors, where square denotes cone-shape tip, circle is for spindle-shape tip, and triangle means breakup at injection nozzle. The phase diagram is obtained with 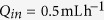 and 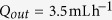. Insets: snapshots of cone-shape tip, spindle-shape tip and breakup at focusing orifice and injection nozzle, respectively.

Figure 3 maps various flow behaviors in Plane (*L*, *l*_*c*_). The cone-shape and spindle-shape tips are separated by *l*_*c*_/*L* = 1. Breakup at the focusing orifice lies above the boundary of *l*_*c*_ = 0.5 *L*, while breakup at the injection nozzle is constrained by *l*_*c*_ < 0.5 *L*. To understand this phase map, we introduce a ratio of the representative length for expansion 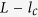 to that for contraction 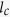, 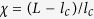, which increases with *L*. The larger 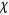 corresponds to stronger diverging flow but weaker converging flow. Initially, 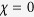 for small values of *L*, indicating a converging flow at the focusing orifice without diverging. As such, shear stress at the focusing orifice is large enough to drag the inner tip into cone-shape, featured by 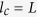 (Fig. 3). As 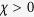, the flow first diverges and then converges, so the tip of inner flow is spindle-shape, represented by 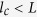 in Fig. 3. In the range of 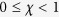, droplet breakup occurs at the focusing orifice, and transitions of droplet breakup mode can be observed with an increase in *L*. When diverging flow magnifies further with the increase of *L*, droplet breaks up at the injection nozzle with 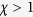. Therefore, the boundary for the transition of breakup location from focusing orifice to injection nozzle reads 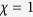, which results in 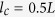 (Fig. 3).

### Condition for the transition of droplet breakup location

To determine the condition for breakup location shifting from the focusing orifice to injection nozzle, we exploit the local capillary number at the injection nozzle in the form of 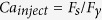, with *F*_*s*_ and *F*_*γ*_ being the total shear forces and capillary forces, respectively. Firstly, we estimate *F*_*s*_ exerted on the inner fluid tip based on a modified Stokes’ drag^32^,^40^,^41^,


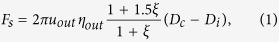


where 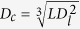 is the characteristic diameter of inner liquid tip, 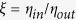 is viscosity ratio, and 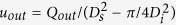 is the mean velocity of outer fluid at the injection nozzle. Capillary forces are evaluated as^32^,^41^


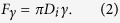


Thus, the local capillary number at the injection nozzle is


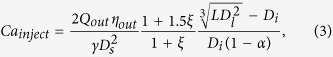


where 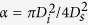 is the ratio of cross-sectional area of the injection nozzle to that of the square capillary. Droplet breakup at the injection nozzle occurs only when 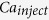 exceeds a critical value 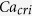. Eq. (3) indicates that 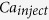 increases with increasing *L*, so 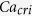 implies a critical *L* for the transition to occur. The critical *L* depends on fluid properties, channel dimensions, and flow rates.

Now we determine the critical *L* triggering the breakup at the injection nozzle based on Eq. (3). The dynamic dimension 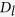 is found to be proportional to orifice distance *L* (see supplementary Fig. S1), thereby it is reasonable to estimate 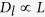. Assuming constant 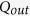, 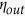, 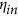, 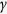 and 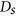 in Eq. (3), we have 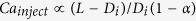. Therefore, the condition for droplet breakup at the injection nozzle, 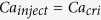, leads to


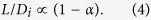


**Figure 4:**
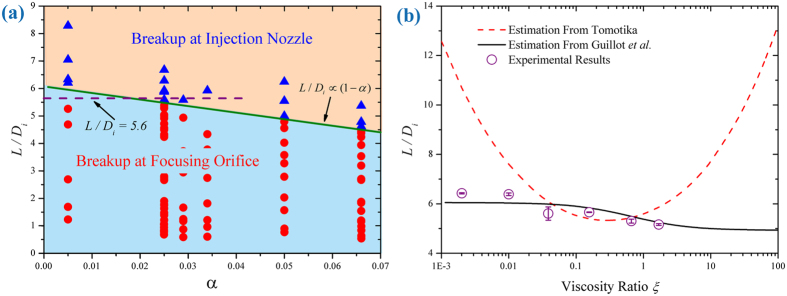
Condition for the transition of droplet breakup location. (**a**) Critical 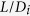 for breakup at injection nozzle as a function of 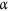. Solid line represents the condition of 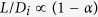 from Eq. (4) where 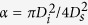, while dashed line denotes 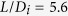 as an estimate under the assumption of 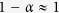 with 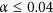. For the same *α*, several values of *D*_*f*_ are tested (see supplementary Fig. S2). Triangle represents breakup at injection nozzle, while circle denotes breakup at focusing orifice. Data are obtained with viscosity ratio 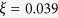. (**b**) Plot of 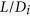 as a function of viscosity ratio 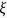. The black solid curve is obtained from Eq. (7) based on the result of Guillot *et al.*^42^, while the red dashed curve is estimated from Tomotika^46^ (see “determining the most unstable mode of a viscous jet” in supplementary information). Experimental results show better agreement with solid-curve estimation. Since the dashed-curve is taken under the circumstance of unbounded quasi-static flow, while solid-curve considers device confinement and flow rates, the difference in the two estimates reveals the significance of channel confinement and flow rates in affecting confined droplet breakup.

Eq. (4) shows that the condition is represented as the ratio of orifice distance *L* to the outer diameter of the injection nozzle *D*_*i*_, 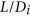, which decreases linearly with 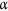. We confirm this relation experimentally as the solid line in Fig. 4a.

In subsequent analysis, we examine the analytic solution for condition 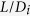, which can be achieved by taking all the influencing parameters into consideration and making a quantitative estimation of 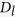. Practically, 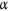 is much smaller than unity, (for example 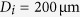 gives 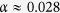), so we assume that 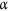 is small enough to assume 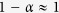 in the following analysis. According to Rayleigh-Plateau instability, the most unstable mode for the jet breakup gives 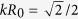
^ ^42,43, where 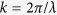 is the wave number with wavelength approximated as 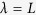 in our case. 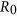 is the unperturbed jet radius and estimated as 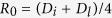, which is the average radius of the injection nozzle and the maximum tip. Replacing *k* and 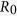 by 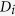, 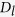 and *L*, we get,


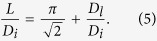


Eq. (5) provides an accurate estimation of the relation between 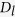 and *L* compared with the previous simplified one 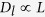. Since breakup at the injection nozzle occurs when 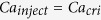, the following relation is achieved by rewriting Eq. (3) with the assumption of 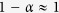,


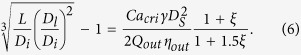


Solving Eqs. (5) and (6) together, 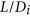 is finally obtained in the following form


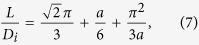


with 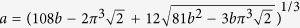, and 
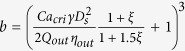
.

Eq. (7) is achieved under two assumptions: 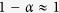, and neglecting the influence of focusing orifice (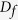). Our experimental results validate these assumptions. For experimental cases with 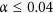 (guaranteeing 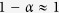) but different 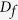 values, the critical condition 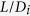 basically keeps constant as 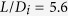 (dashed line in Fig. 4a, also see supplementary Fig. S2), independent of 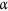. However, a determination of 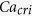 is necessary to predict 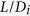 theoretically.

As presented by Erb *et al.*^32^, a 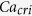 value of 0.1 is accurate enough to predict the condition for droplet breakup over a wide range of viscosity ratios. We thus plot 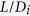 as a function of 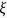 by using 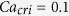. The other parameters involved are 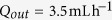, 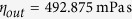, 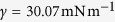, 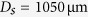, respectively. As shown in Fig. 4b, the prediction (solid line) agrees well with experimental results when viscosity ratio varies over three orders of magnitude.

### Characteristics of tip-multi-breaking mode

Having determined the condition for droplet breakup at the injection nozzle, we now turn our attention to breakup at the focusing orifice. According to Taylor^44^, we define outer phase capillary number as 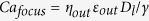 locally at the focusing orifice, where 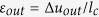 is the strain rate represented by the velocity gradient along the flow direction. 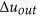 is estimated as the difference between the average flow velocity at the focusing orifice, 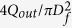, and that at the maximum tip diameter, 
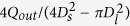
, which yields 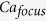 in the following form


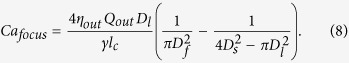


As contraction length 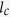 increases, capillary number 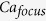 decreases, indicating a weaker external shear stress. Since increasing distance *L* results in larger contraction length 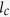 (Fig. 3), capillary number 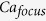 decreases with *L*. Eq. (8) thus accounts for the transition from dripping to geometry-controlled mode by increasing *L*. Next, we use 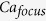 to quantify the droplet breakup in tip-multi-breaking mode at the focusing orifice.

Droplet sizes vary with local capillary number. For dripping, a scaling law^45^ suggests 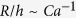, with *Ca* being capillary number, *R* being droplet radius, and *h* being channel dimension. However, for tip-multi-breaking mode, such a scaling cannot hold, because droplet sizes are not uniform even for the same capillary number (Fig. 2). In this case, droplet size should be normalized by a dynamic length scale instead of a static one. In tip-multi-breaking mode, force balance between shear stress and surface tension gives 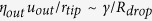, where *R*_*drop*_ and *r*_*tip*_ are radii of the droplet and tip neck, respectively. If the characteristic velocity 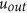 is approximated as 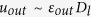 by considering geometry parameters, then the force balance gives 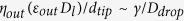 with *R*_*drop*_ and *r*_*tip*_ replaced by *D*_*drop*_ and *d*_*tip*_. Finally, we arrive at a scaling law,


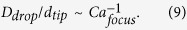


**Figure 5:**
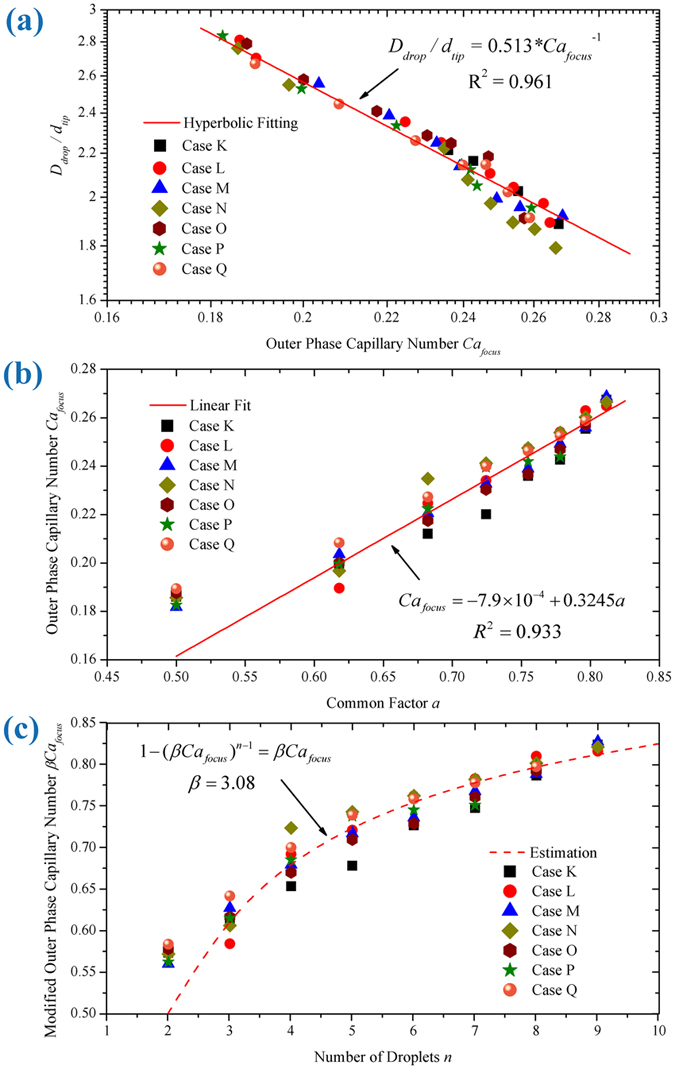
Characteristics of tip-multi-breaking mode. (**a**) Log-log plot of droplet size as a function of local capillary number *Ca*_*focus*_. Experimental data collapse onto a fitted line of 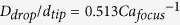. (**b**) Outer phase capillary number *Ca*_*focus*_ showing a linear relation to common ratio *a*. (**c**) Plot of capillary number *Ca*_*focus*_ *versus* droplet number *n*. Dashed line shows the estimation of 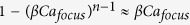 (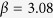), which gives a fair prediction when 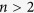, and the larger the value of *n* is, the smaller the deviation becomes. Different symbols denote different cases (Table 1).

Eq. (9) provides a scaling for tip-multi-breaking in a form similar to 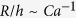, but different in that droplet diameter *D*_*drop*_ is scaled by a dynamic length *d*_*tip*_, rather than the static channel dimension *h*. This scaling is confirmed to agree with the experimental data very well, as shown in Fig. 5a. For a droplet sequence with polydisperse droplets (Fig. 2), 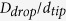 equals constant due to the same capillary number 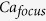. So the non-uniformity of the droplets is interpreted by the change of inner tip diameter *d*_*tip*_. For example, descending size distribution of tip-multi-breaking mode in Fig. 2a is the result of 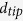 thinning monotonically with time during the formation of one droplet sequence. Likewise, different size distributions in Fig. 2b,c are attributed to the different ways in which 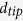 alters with time.

Apart from influencing droplet size distribution in tip-multi-breaking mode, capillary number 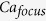 affects the number of droplets in the sequence as well. Here, we focus only on the droplet sequence with descending size distribution, as shown in Fig. 2a. Previously, we found that droplet number *n* qualitatively increases with capillary number 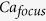
37. Now, to obtain a quantitative relationship between *n* and 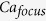, it is necessary to summarize here some fundamental results from ref. 37. First, the individual droplet size in one droplet sequence constitutes a geometrical progression, with common factor being *a*. Second, common factor *a* and droplet number *n* are related as


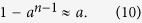


If *a* and 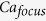 are related, then the relation between *n* and 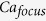 would finally be obtained based on Eq. (10). Experimentally, common ratio *a* is found to increase linearly with 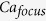, 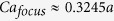 (Fig. 5b). Then, by assuming 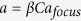 (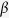 is a constant), we get


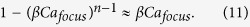


When constant 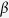 fits as 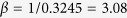, experimental data basically collapse around the prediction given by Eq. (11) (Fig. 5c). The discrepancy between the prediction and experimental data gets smaller and smaller when *n* grows. In fact, 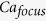 varies smoothly, while *n* are discrete natural numbers (*n* > 1). So for every single *n*, there should be a narrow range rather than only one value of 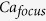, as displayed in Fig. 5c. Based on Eq. (11) droplet train with prescribed droplet number can be tuned on-demand by varying the matching capillary number, for instance by changing outer phase flow rate. These droplet sequences may have potential applications in materials science, for example, in designing new barcode emulsions and particles with multiple cores of different sizes and numbers.

### Oscillation of the inner liquid tip

**Figure 6:**
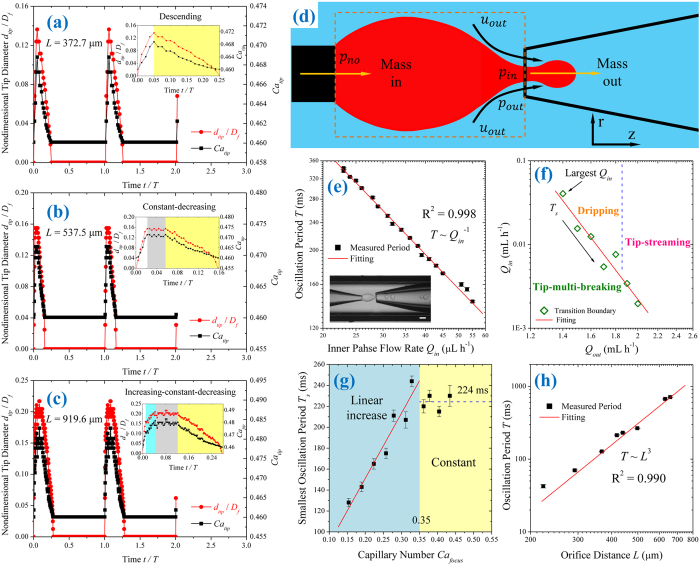
Oscillation of inner liquid tip. (**a**–**c**) Evolutions of nondimensional tip diameter *d*_*tip*_/*D*_*f*_ (red) and capillary number *Ca*_*tip*_ (black) at the focusing orifice in tip-multi-breaking mode. *D*_*f*_ = 210 μm. (**a**) Descending behavior of *d*_*tip*_/*D*_*f*_ and *Ca*_*tip*_ measured from Fig. 2a with *L* = 372.7 μm, and period *T* = 160 ms. (**b**) Constant-decreasing behavior of *d*_*tip*_/*D*_*f*_ and *Ca*_*tip*_ measured from Fig. 2b with *L* = 537.5 μm, and period *T* = 456 ms. (**c**) Increasing-constant-decreasing behavior of *d*_*tip*_/*D*_*f*_ and *Ca*_*tip*_ measured from Fig. 2c with *L* = 919.6 μm, and period *T* = 834 ms. The insets in (**a**–**c**) display the magnifications of tip evolution and temporal variation of *Ca*_*tip*_. Since no droplet is generated during the initial period of increasing tip diameter (white area), we focus on the later stages of tip-diameter increasing (cyan area), constant tip-diameter (gray area) and tip-diameter decreasing (yellow area) where droplets are generated in (a–c). (**d**) Schematic of the control volume of the inner liquid tip confined by the left injection nozzle and right focusing orifice. *p*_*in*_ and *p*_*out*_ are pressures at the focusing orifice for inner and outer phases, respectively, while *p*_*no*_ is the inner fluid pressure at the injection nozzle. (**e**) Log-log plot of oscillation period *T versus* inner fluid flow rate *Q*_*in*_, with *Q*_*out*_ = 1.5 mL h^−1^. Inset: snapshot of a droplet-sequence generation. Scale bar, 200 μm. (**f**) Transition boundary of tip-multi-breaking mode in *Q*_*out*_-*Q*_*in*_ plane; data adapted from ref. 37. (**g**) Minimum oscillation period *T*_*s*_ *versus* capillary number *Ca*_*focus*_. (**h**) Log-log plot of oscillation period *T versus* Orifice distance *L* for fixed inner and outer flow rates. *Q*_*in*_ = 4.5 μL h^−1^, *Q*_*out*_ = 3 mL h^−1^. The device used in (**e**,**g**,**h**) is case *L* with geometrical dimension shown in Table 1.

The periodic oscillation of the tip features tip-multi-breaking mode. We show the evolution of *d*_*tip*_ for three types of tip behaviors, descending, constant-decreasing, and increasing-constant-decreasing in Fig. 6a–c, respectively, from experimental data. To quantify the variation of outer-fluid viscous stress during the *d*_*tip*_ evolution, we define its local capillary number *Ca*_*tip*_ by *Ca*_*tip*_ = 4*η*_*out*_*Q*_*out*_/[*π*(*D*_*f*_^2^−*d*_*tip*_^2^)*γ*] (*η*_*out*_ = 492.9 mPa s, *Q*_*out*_ = 3.5 mL h^−1^, *D*_*f*_ = 210 μm and *γ* = 30.07 mN m^−1^ in our experiments) at the focusing orifice, and plot the temporal variation of *Ca*_*tip*_ in Fig. 6a–c. Due to the penetration of inner tip into the focusing orifice (see supplementary movie S2), a *d*_*tip*_-increasing stage occurs at the very beginning of its evolution (white areas in the insets in Fig. 6a–c). No droplet is generated at this initial tip-growing stage because of the low viscous shear from the outer fluid. Afterwards, the droplet-generation takes place as the shear stress is large enough (cyan, gray and yellow areas in the insets in Fig. 6a–c). During the time period of droplet generation, the droplet size, *d*_*tip*_ and *Ca*_*tip*_ all vary in the form of descending (Fig. 6a), constant-decreasing (Fig. 6b), or increasing-constant-decreasing (Fig. 6c) as *L* increases.

Although the flow rates of both inner and outer fluids, the density-weighted area average of local flow field, are kept constant by syringe pumps, the local flow field can be unsteady around the focusing orifice, which induces the oscillation of the inner-fluid tip (Fig. 6a–c). To isolate the mechanism responsible for the tip oscillation, consider the normal stresses balance across the liquid-liquid interface^40^ at the focusing orifice (Fig. 6d):


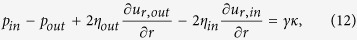


where *p* is pressure, 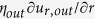 and 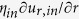 are, respectively, the outer and inner viscous stresses normal to the interface, *κ* is twice the mean curvature of the interface, estimated as 2/*d*_*tip*_ at the focusing orifice. Because the viscosity ratio is much smaller than unity (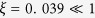), 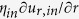 is negligible compared with 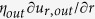. At fixed flow condition (*Q*_*in*_, *Q*_*out*_ and *L* are constant for every single case in Fig. 6a–c), *p*_*out*_ and 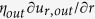 can be assumed to be invariant during the tip thinning. Therefore, according to Eq. (12), *p*_*in*_ increases with the shrinkage of *d*_*tip*_, for which *κ* is increased. When *p*_*in*_ is sufficiently large to compete with the pressure at the nozzle *p*_*no*_ (Fig. 6d), the inner tip is pushed upstream out of the focusing orifice. After recoiling, the tip is inflated by the inner fluid flow again and penetrates into the focusing orifice once it is large enough. The variation in inner pressure *p*_*in*_ is thus responsible for the tip oscillation. Further studies are needed to quantify this force analysis by experimentally measuring local pressure and flow fields inside microchannels, which is beyond our current capability of experiments.

The three distinct types of tip oscillation (Fig. 6a–c) correspond to different values of *L*. Eq. (12) accounts for this *L*-dependent behavior of *d*_*tip*_. *Q*_*in*_ and *Q*_*out*_ are held constant in Fig. 6a–c, so that *p*_*in*_ and *p*_*out*_ can be assumed as constant at the maxima of *d*_*tip*_. With enlarging *L*, the reduction in 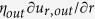 leads to a decrease in the mean curvature *κ*. Consequently, as *κ* = 2/*d*_*tip*_, the maxima of *d*_*tip*_ increases with *L*, as confirmed experimentally in Fig. 6a–c. For the largest *L* in Fig. 6c, the tip needs the longest time to fully develop into its maximal diameter *d*_*tip*_ after penetrating into the focusing orifice. An increasing stage of *d*_*tip*_ is therefore identified (cyan area in the inset in Fig. 6c). Afterwards, *d*_*tip*_ is temporarily stabilized (gray area) because of the transient mass balance of mass in and out from the tip (Fig. 6d), followed by the necking thinning (yellow area) due to inner fluid drainage (see supplementary Fig. S3 for experimental confirmation). Due to different time required for the tip to be fully developed, intermediate *L* in Fig. 6b holds *d*_*tip*_ constant for a while before tip thinning (inset in Fig. 6b), whereas the case with the smallest *L* in Fig. 6a has *d*_*tip*_ diminishing immediately once the tip diameter approaches the peak (inset in Fig. 6a).

We now show the variation of tip oscillation period *T* with inner flow rate *Q*_*in*_, outer capillary number *Ca*_*focus*_, and orifice distance *L*. In determining the relationship between *T* and *Q*_*in*_, *Q*_*out*_ and *L* are fixed as constant. Since droplet sequence is fixed by *Ca*_*focus*_, in this case, the volume *V*_*s*_ of one droplet sequence is invariant when *Q*_*in*_ varies. Thus, mass conservation, *Q*_*in*_ = *V*_*s*_/*T*, suggests that *T* is inversely proportional to *Q*_*in*_, verified by experiments in Fig. 6e. As *Q*_*out*_ changes, there is a smallest oscillation period *T*_*s*_ corresponding to the largest *Q*_*in*_ that enables the occurrence of tip-multi-breaking mode (Fig. 6f), as found in ref. 37. We show variation of *T*_*s*_ with *Ca*_*focus*_ in Fig. 6g where *T*_*s*_ is experimentally measured on the transition boundary between the tip-multi-breaking mode and the others shown in Fig. 6f. It shows that, for capillary number below 0.35, *T*_*s*_ increases linearly with *Ca*_*focus*_. For *Ca*_*focus*_ above 0.35, *T*_*s*_ is however essentially independent of capillary number, and fluctuates around 224 ms in our experiments. This is due to the volume reduction of the tip as *Ca*_*focus*_ increases (see supplementary Fig. S4 for details). With both *Q*_*in*_ and *Q*_*out*_ fixed, an increase in *L* increases the tip volume *V*_*t*_ (dashed box in Fig. 6d) because *V*_*t*_ *~* *LD*_*l*_^2^ *~* *L*^3^. As the volume *V*_*s*_ of one droplet sequence is proportional to the tip volume *V*_*t*_, *V*_*s*_ *~* *V*_*t*_ *~* *L*^3^; the mass conservation *Q*_*in*_ = *V*_*s*_/*T* leads to *T* *~* *V*_*s*_ *~* *L*^3^ when *Q*_*in*_ is kept constant. This is experimentally confirmed in Fig. 6h.

## Concluding Remarks

In conclusion, we have systematically studied the influence of expansion-contraction geometry on droplet breakup in capillary microfluidic devices, which we separate into two parts: at the injection nozzle where flow diverges, and at the focusing orifice where flow converges. We demonstrate that the variation of expansion-contraction dimension, tuned by orifice distance *L*, affects two local shear stresses at the focusing and injection orifices, and thus significantly influences the flow behavior. These influences include changes of droplet breakup location and breakup mode. At the injection nozzle, we derive a condition of critical *L* for breakup location transition by balancing local shear and capillary forces. Similarly, at the focusing orifice, we determine the local capillary number as a ratio of shear stresses to capillary effects, and quantify its relation to the size and number of droplets in tip-multi-breaking mode. The force balance on the liquid-liquid interface at the focusing orifice provides physical insight into the dynamical behavior of the tip oscillation. We have also experimentally examined the variation of the tip oscillation period with inner fluid flow, outer phase capillary number and orifice distance. Beyond the capillary devices used in this work, we expect our results to be also applicable to other two-phase microsystems involving expansion-contraction structures. Such fundamental understanding of droplet breakup in microfluidics involving expansion-contraction geometries would be useful in droplet generation, manipulation, and microfluidic device design.

## Additional Information

**How to cite this article**: Zhu, P. *et al.* Droplet Breakup in Expansion-contraction Microchannels. *Sci. Rep.* **6**, 21527; doi: 10.1038/srep21527 (2016).

## Supplementary Material

Supplementary Movie 1

Supplementary Movie 2

Supplementary Information

## Figures and Tables

**Table 1:** Eighteen cases tested with different device geometries.

Case Number	*D*_*i*_ (μm)	*D*_*f*_ (μm)	*L*(μm)	Case Number	*D*_*i*_ (μm)	*D*_*f*_ (μm)	*L*(μm)
A	86	258	97–714	K	147	242	806
B	186	258	197–1100	L	147	242	267
C	203	258	119–1136	M	147	242	570
D	217	258	130–1284	N	147	242	150
E	186	150	126–1166	O	147	176	272
F	186	210	146–1094	P	73	242	270
G	186	353	155–1038	Q	208	242	265
H	265	155	1208–1654	L	147	197	409
I	305	155	163–1410				
J	305	258	192–1263				

Cases A–J, with various combinations of *D*_*i*_ and *D*_*f*_, are conducted to investigate the condition for the location shift of droplet breakup from focusing orifice to injection nozzle. For each case from A to J, the orifice distance *L* is variable, starting from an initial value comparable to the diameter of injection nozzle *D*_*i*_, ending with the value when droplet breakup occurs at the injection nozzle. The range of *L* for each case from A to J can also be found in Fig. 3. Cases K–Q are used to characterize the influence of device geometry on tip-multi-breaking mode. Different combinations of *D*_*i*_, *D*_*f*_ and *L* are studied. Case *L* is used to develop the correlation between the oscillation period *T* and the capillary number *Ca*_*focus*_ in tip-multi-breaking mode. The capillary number *Ca*_*focus*_ ranges from 0.15 to 0.45 in our experiments.
