# Supplementary material for: Droplet Breakup in Expansion-contraction Microchannels
Source: Sci Rep. 2016 Feb 22;6:21527. doi: 10.1038/srep21527 (PMC4761913; doi:10.1038/srep21527)
Supplement: Supplementary Information [file srep21527-s3.pdf]

Supplementary Information

"Droplet Breakup in Expansion-contraction Microchannels"

Pingan Zhu[1,2], Tiantian Kong[1,2], Leyan Lei[1,2], Xiaowei Tian[1,2], Zhanxiao Kang[1,2] and Liqiu Wang[1,2,]*

[1]Department of Mechanical Engineering, the University of Hong Kong, Hong Kong

[2]HKU-Zhejiang Institute of Research and Innovation (HKU-ZIRI), 311300, Hangzhou, Zhejiang, China

* Corresponding author: lqwang@hku.hk

This file includes:

1. Legends of Supplementary Movies S1 and S2
2. Confirming Constant Inner Flow Rate
3. Determining the Most Unstable Mode of a Viscous Jet
4. Supplementary Figures
5. Supplementary Tables

1. Legends of Supplementary Movies S1 to S2

1.1 Supplementary Movie S1:

Tip Morphology Transformation and Breakup at Injection Nozzle

This video demonstrates the transformation of inner liquid tip with enlarging orifice distance *L* and the occurrence of breakup at injection orifice in a capillary microfluidic device. The inner phase is a mixture of 70 wt.% glycerol and 30 wt.% distilled water, and outer phase is silicone oil. The inner and outer phase flow rates are 0.5 mL h^{-1} and 3.5 mL h^{-1}, respectively. The video is recorded with 500 frames per second (fps) and played with 10 fps.

1.2 Supplementary Movie S2:

Droplet Size Distribution Affected by Orifice Distance *L*

This video shows three different size distributions in tip-multi-breaking mode influenced by orifice distance *L*. Droplets are generated sequence by sequence periodically in this situation. The inner phase is a mixture of 70 wt.% glycerol and 30 wt.% distilled water, and outer phase is silicone oil. The inner and outer phase flow rates are 0.02 mL h^{-1} and 3.5 mL h^{-1}, respectively. The video is recorded with 500 fps and played with 10 fps.

2. Confirming Constant Inner Flow Rate

To confirm the constant inner flow rate in our experiments, the inner and outer flow rates are set by syringe pumps with Q_{in} = 4.5 μL h^{-1} and Q_{out} = 3 mL h^{-1}, respectively. The channel dimension are D_f = 197 μm and D_i = 147 μm. Various droplet generation processes are obtained with the increase of orifice distance L (Fig. S5).

To estimate the inner flow rate by analyzing the captured video, we build a general model to calculate the volume of droplets in one sequence (Fig. S6). The three types of droplet size distribution (descending, constant-decreasing, and increasing-constant-decreasing) are distinguished by the values of n_1, n_2 and b (Fig. S6 and Table S2). Considering the symmetry, we argue that $\theta_1 = \theta_2$ for droplet sequence of increasing-constant-decreasing mode.

For the left and right sequences, we assume that droplet size obeys a geometrical progression, with the first droplet size $R_1 = R$. So we have

$$R_i = Ra^{n-1}, \tag{1}$$

for both of the left and right sequences. Common factor is calculated by $a = (1 - \sin\theta)/(1 + \sin\theta)$[1]. Therefore, the volumes of droplets for left and right sequences are

$$V_k = \frac{4}{3}\pi R^3 \frac{1-{a_k}^{3n_k}}{1-{a_k}^3}. \tag{2}$$

Subscript "k" can be either "1" or "2", indicating the left and right sequence, respectively. For the middle part with constant droplet size distribution, the volume is calculated as follows:

$$V_m = \frac{4}{3}\pi R^3 b. \tag{3}$$

Thus, the total volume for one droplet sequence gives as follows,

$$V_s = V_k + V_m = \frac{4}{3}\pi R^3 \left(\frac{1-{a_1}^{3n_1}}{1-{a_1}^3} + b + \frac{1-{a_2}^{3n_2}}{1-{a_2}^3}\right). \tag{4}$$

Accordingly, the inner flow rate Q_{in} is estimated by the following equation:

$$Q_{in} = V_s/T, \tag{5}$$

with T being the period of the droplet sequence generation. All the measurements and related calculations can be found in Table S2.

We plot the results of Q_{in} versus L in Fig. S7. It suggests that within the tested range of L, inner flow rate is fairly constant, bounded in the range of 4 μL h^{-1} to 5 μL h^{-1}, consistent with the value monitored by syringe pump of 4.5 μL h^{-1}.

3. Determining the Most Unstable Mode of a Viscous Jet

Here we consider that an initial infinitesimal perturbation grows on a long cylindrical viscous jet with viscosity η_{in} immersed in another viscous liquid with viscosity η_{out}, and the growing perturbation is proportional to $e^{i(\omega t+kz)}$. According to Tomotika[2], the dispersion relation gives,

$$i\omega=\frac{\gamma}{2R_0\eta_{out}}(1-x^2)\Phi(x,\xi)\,, \tag{6}$$

where ω is frequency of the perturbation, γ the interfacial tension, R_0 the radius of the unperturbed jet. $x=kR_0$ is the dimensionless wave number, where $k=2\pi/\lambda$ is wave number with λ being the wavelength of the perturbation. $\Phi(x,\xi)$ is a function of x and viscosity ratio ξ ($\xi=\eta_{in}/\eta_{out}$) in the form of

$$\Phi(x,\xi)=\frac{N(x,\xi)}{D(x,\xi)}\,, \tag{7}$$

with

$$N(x,\xi)=I_1(x)\Delta_1-\left[xI_0(x)-I_1(x)\right]\Delta_2\,, \tag{8}$$

$$\begin{aligned}D(x,\xi)=\xi\left[xI_0(x)-I_1(x)\right]\Delta_1-\xi\left[(x^2+1)I_1(x)-xI_0(x)\right]\Delta_2\\ -\left[xK_0(x)+K_1(x)\right]\Delta_3-\left[(x^2+1)K_1(x)+xK_0(x)\right]\Delta_4\end{aligned}\,. \tag{9}$$

In Eqs. (8) and (9), $I(x)$ and $K(x)$ are modified Bessel Functions, with subscripts "0" and "1" representing the order. In addition, Δ_1, Δ_2, Δ_3 and Δ_4 are functions of x and ξ, given in the following form:

$$\Delta_1=\begin{vmatrix} xI_0(x)-I_1(x) & K_1(x) & -xK_0(x)-K_1(x)\\ I_0(x)+xI_1(x) & -K_0(x) & -K_0(x)+xK_1(x)\\ \xi xI_0(x) & K_1(x) & -xK_0(x)\end{vmatrix}\,, \tag{10}$$

$$\Delta_2=\begin{vmatrix} I_1(x) & K_1(x) & -xK_0(x)-K_1(x)\\ I_0(x) & -K_0(x) & -K_0(x)+xK_1(x)\\ \xi I_1(x) & K_1(x) & -xK_0(x)\end{vmatrix}\,, \tag{11}$$

$$\Delta_3=\begin{vmatrix} I_1(x) & xI_0(x)-I_1(x) & -xK_0(x)-K_1(x)\\ I_0(x) & I_0(x)+xI_1(x) & -K_0(x)+xK_1(x)\\ \xi I_1(x) & \xi xI_0(x) & -xK_0(x)\end{vmatrix}\,, \tag{12}$$

$$\Delta_4 = \begin{vmatrix} I_1(x) & xI_0(x)-I_1(x) & K_1(x) \\ I_0(x) & I_0(x)+xI_1(x) & -K_0(x) \\ \xi I_1(x) & \xi x I_0(x) & K_1(x) \end{vmatrix}. \qquad (13)$$

The most unstable mode corresponds to $i\omega$ with the biggest value, and $i\omega$ is proportional to $(1-x^2)\Phi(x,\xi)$ according to Eq. (6). Now let

$$F(x,\xi) = (1-x^2)\Phi(x,\xi), \qquad (14)$$

so for a situation with viscosity ratio ξ fixed, the most unstable jet matches the value x_0 that yields the maximum $F(x_0,\xi)$. Since $F(x,\xi)$ has only one maximum value in the whole domain of x, as shown in Fig. S8, we find the maximum of $F(x,\xi)$ when $\frac{\partial F(x,\xi)}{\partial x}|_{x_0} = 0$, namely

$$-2x\frac{N(x,\xi)}{D(x,\xi)} + (1-x^2)(\frac{1}{D(x,\xi)}\frac{\partial N(x,\xi)}{\partial x} - \frac{N(x,\xi)}{D^2(x,\xi)}\frac{\partial D(x,\xi)}{\partial x}) = 0. \qquad (15)$$

Solving Eq. (15) in MATLAB, we obtain the value of $x_{0(\xi)}$. We thus have, by using $x = kR_0$ and $k = 2\pi/\lambda$,

$$\frac{\lambda}{2R_0} = \frac{\pi}{x_{0(\xi)}}. \qquad (16)$$

Letting orifice distance $L = \lambda$, and injection orifice diameter $D_i = 2R_0$, we estimate

$$\frac{L}{D_i} = \frac{\pi}{x_{0(\xi)}} \qquad (17)$$

as the condition for the occurrence of breakup at injection nozzle. Eq. (17) is plotted in the $L/D_i - \xi$ plane with $x_{0(\xi)}$ solved in Eq. (15), shown in Fig. S9.

References

1 Zhu, P. A., Kong, T. T., Kang, Z. X., Tian, X. W. & Wang, L. Q. Tip-multi-breaking in capillary microfluidic devices. *Sci. Rep.* **5**, doi:10.1038/srep11102 (2015).

2 Tomotika, S. On the instability of a cylindrical thread of a viscous liquid surrounded by another viscous fluid. *Proc. R. Soc. Lond. A* **150**, 322-337 (1935).

4. Supplementary Figures

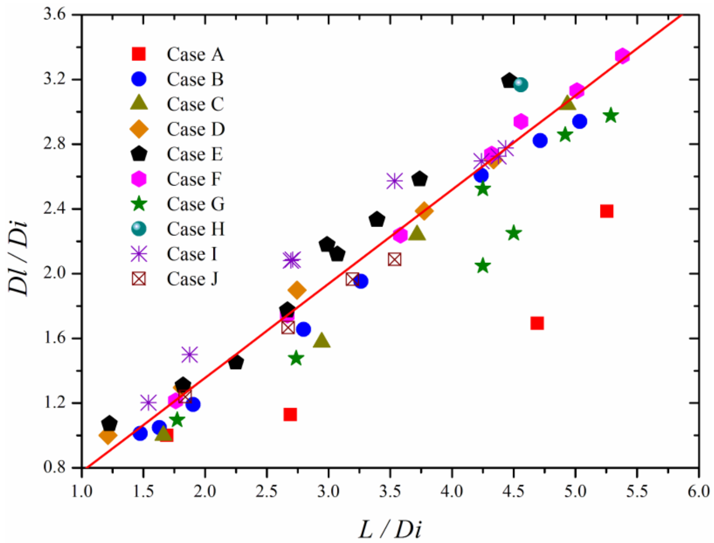

Figure S1. Plot of D_l *versus* L. All the data collapse around the fitted straight line, indicating a linear relation between D_l and L. D_l and L are both normalized by D_i. Q_{in} = 0.5 mL h^{-1}, Q_{out} = 3.5 mL h^{-1}.

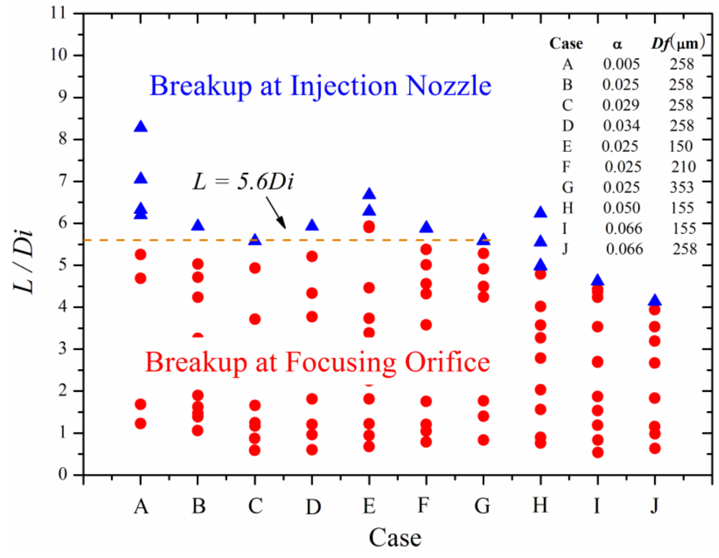

Figure S2. Condition of L / D_i for cases A-J used in experiments. When α is less than 0.04 (Cases A-G), the critical condition L / D_i is approximated as a constant value, independent of α and the diameter of focusing orifice D_f. $L / D_i \approx 5.6$ is obtained experimentally with viscosity ratio $\xi = 0.039$.

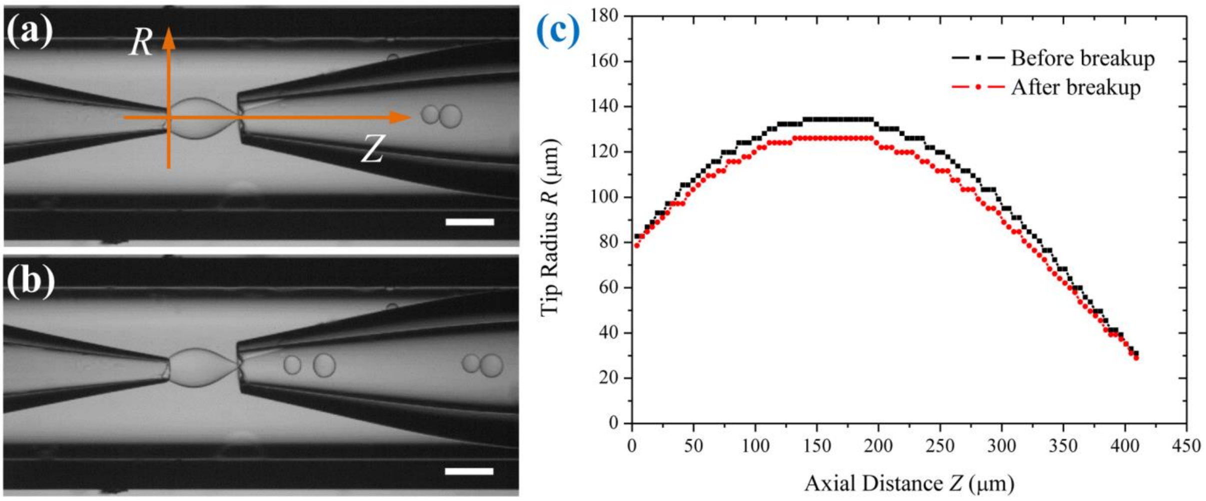

Figure S3. Change in the liquid tip volume during one sequence of droplets breakup. (**a**) Time instant just before droplet breakup. The tip radius *R versus* axial distance *Z* is shown in (c). (**b**) Time instant after the droplet sequence generation. Time interval between (a) and (b) is 60 ms. (**c**) Plot of tip radius *versus* axial distance. The black curve is the contour for the tip in (a), while the red one is for (b). Apparently, the lower position of the red curve than the black one suggests a drainage of inner fluid in the tip reservoir, which induces the neck thinning and the final retraction of inner tip during one period of droplet sequence formation. The contour is obtained by converting the original images (a) and (b) into binary images, and extracting the coordinate of every pixel point on the tip boundary in MATLAB. Scale bars, 300 μm.

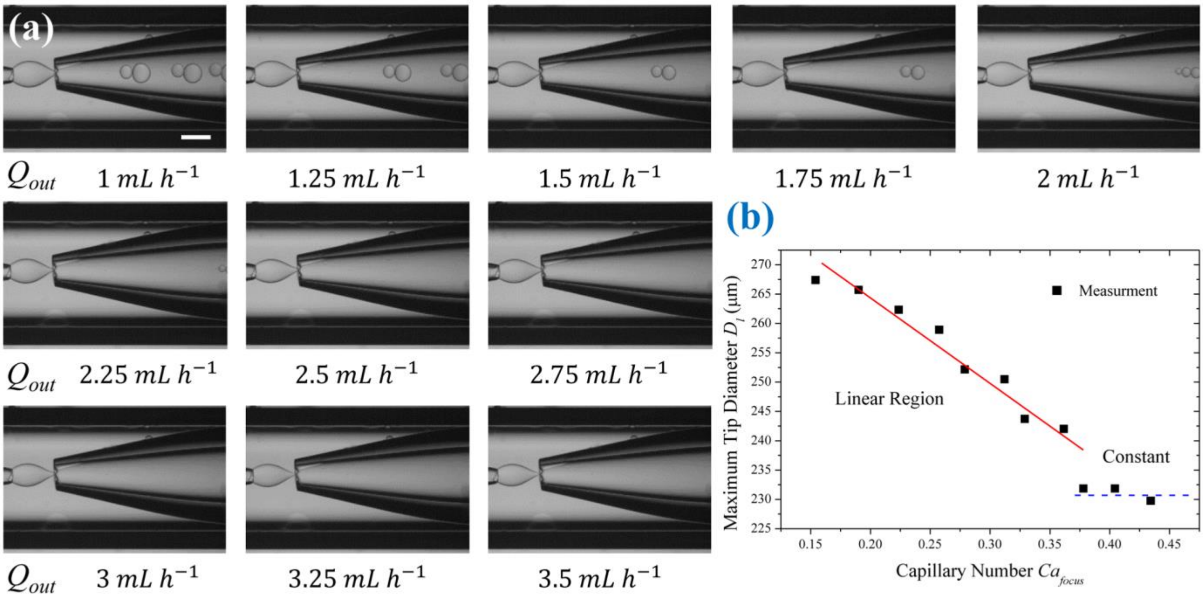

Figure S4. Variation in the tip volume with the increase of capillary number *Ca_{focus}*. (**a**) Snapshots of the tip volume with the increase of outer phase flow rate Q_{out} (the value below each image). The corresponding capillary numbers Ca_{focus} are 0.154, 0.190, 0.224, 0.258, 0.279, 0.312, 0.329, 0.362, 0.378, 0.404 and 0.434 from low to high Q_{out}. Inner flow rates Q_{in} for each snapshot are 135 μL h^{-1}, 90 μL h^{-1}, 48 μL h^{-1}, 45 μL h^{-1}, 20 μL h^{-1}, 15 μL h^{-1}, 10 μL h^{-1}, 9 μL h^{-1}, 5 μL h^{-1}, 4 μL h^{-1} and 2.5 μL h^{-1} with increasing Q_{out}. Every snapshot is captured at the time instant just before the first droplet in the sequence is emitted. (**b**) Plot of maximum tip diameter D_l *versus* capillary number Ca_{focus}. D_l is measured from the snapshots in (a). For capillary number lower than 0.35, D_l decrease monotonically as Ca_{focus} increase. In contrast, for Ca_{focus} larger than 0.35, D_l varies more gently with the increase of Ca_{focus}. Scale bar, 300 μm.

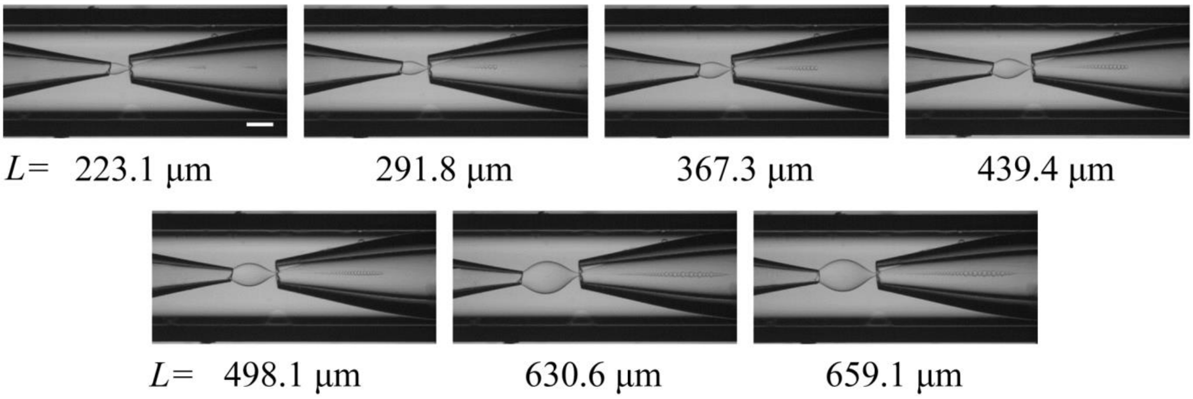

Figure S5. Snapshots of droplet sequence generation with enlarging orifice distance *L*. The value of *L* is provided below each image. Inner and outer flow rates are held constant by the syringe pumps with Q_{in} = 4.5 µL h^{-1} and Q_{out} = 3 mL h^{-1} (the pump we used can achieve controlling flow rate as low as 1 µL h^{-1} by using 1cc Terumo plastic syringe). Droplet size distribution changes from descending, to constant-decreasing, and to increasing-constant-decreasing mode with the enlarging *L*. Scale bar, 300 µm.

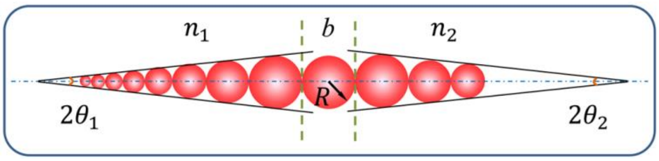

Figure S6. Model for calculating the inner flow rate Q_{in}. The droplet sequence is divided into three parts: the left sequence with droplet numbers n_1 and apex angle $2\theta_1$, the middle part with constant droplet size of number b, and the right sequence with droplet numbers n_2 and apex angle $2\theta_2$. For droplet sequence with "descending" size distribution, $n_2 = b = 0$; for the sequence with "constant-decreasing" size distribution, $n_2 = 0$; for that with "increasing-constant-decreasing" size distribution, none of n_1, n_2 and b is zero. R is the radius of the droplet with constant size, and is also the first droplet size "R_1" for the left and right sequences.

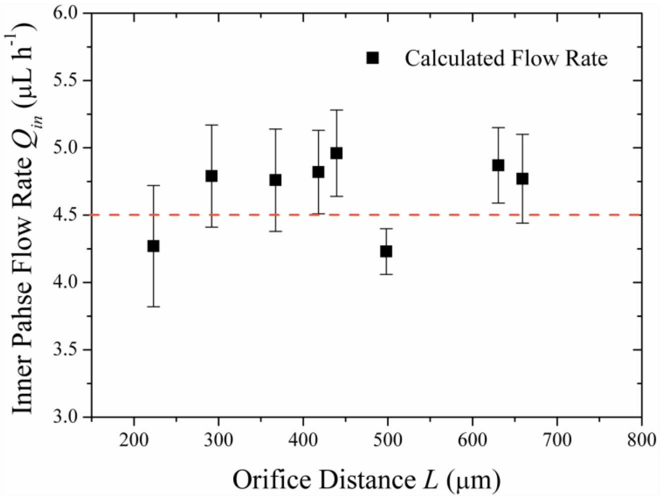

Figure S7. Plot of calculated Q_{in} *versus* orifice distance L. Within the tested range of L, the calculated flow rates lie between 4 μL h^{-1} and 5 μL h^{-1}, consistent with the flow rate of Q_{in} = 4.5 μL h^{-1} set by the syringe pump. This suggests that the inner flow rate in our experiments is constant and controlled by the pump.

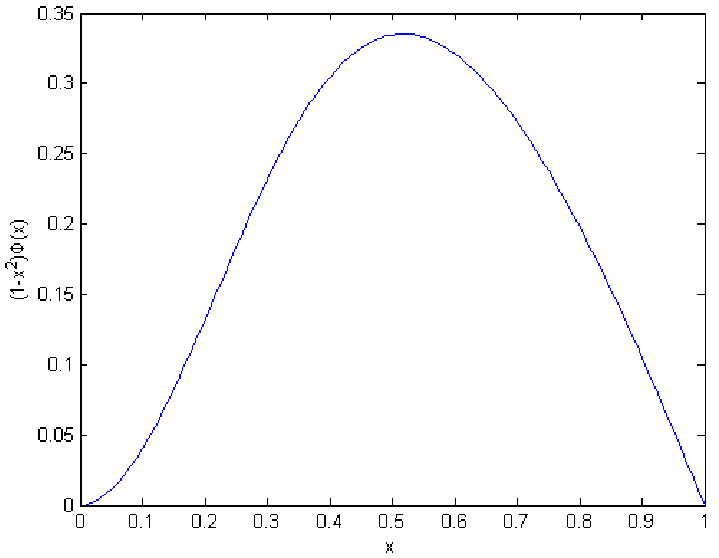

Figure S8. ***F*(*x*, 0.039) as a function of *x* with viscosity ratio *ξ* = 0.039.**

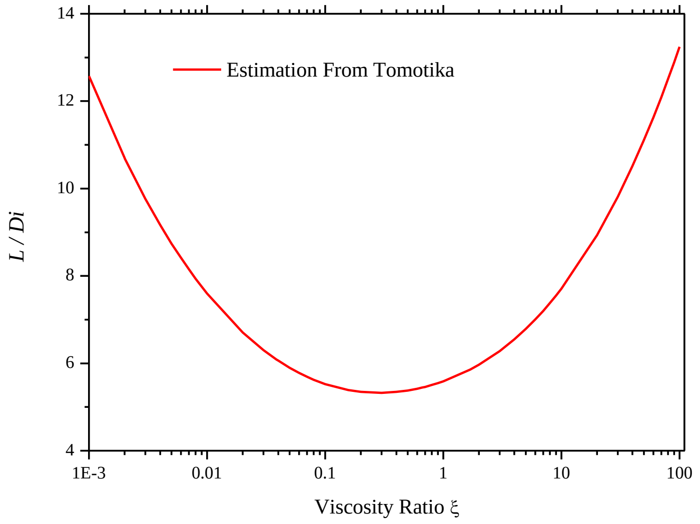

Figure S9. Condition for breakup at injection nozzle estimated from Tomotika.

5. Supplementary Tables

Outer phase fluid		Inner phase fluid		Viscosity ratio $\xi = \eta_{in} / \eta_{out}$
Fluid	Viscosity η_{out} (mPa s)	Fluid	Viscosity η_{in} (mPa s)	
silicone oil	492.9 ± 6.9	pure water	0.992 ± 0.005	0.002
		47 wt.% glycerol	4.982 ± 0.065	0.010
		70 wt.% glycerol	19.07 ± 0.12	0.039
		85 wt.% glycerol	77.38 ± 0.57	0.157
		95 wt.% glycerol	327.4 ± 1.5	0.664
		pure glycerol	831.1 ± 12.5	1.686

Table S1. Material properties of fluids used in experiments. The outer phase fluid is silicone oil with fixed viscosity η_{out}, while the inner fluids are various mixtures of water and glycerol. Different weight percentages of water and glycerol are shown in the table, with different values of viscosity η_{in}. The viscosity ratio of inner and outer fluids is defined as $\xi = \eta_{in} / \eta_{out}$. 70 wt.% glycerol as inner fluid is used for most of the experiments, except when determining the condition L / D_i for droplet breakup at injection nozzle as a function of viscosity ratio ξ (Fig. 4b in the article).

L (μm)	223.1	291.8	367.3	418.0	439.4	498.1	630.6	659.1
Type	De	De	Con-de	Con-de	In-con-de	In-con-de	In-con-de	In-con-de
n_1	9	12	17	13	27	29	38	42
θ_1	5.662 °	4.805 °	3.530 °	4.565 °	2.350 °	2.034 °	1.533 °	0.851 °
a_1	0.8204	0.8454	0.8840	0.8526	0.9212	0.9314	0.9479	0.9707
n_2	0	0	0	0	4	5	10	13
θ_2	0	0	0	0	2.350 °	2.034 °	1.533 °	0.851 °
a_2	0	0	0	0	0.9212	0.9314	0.9479	0.9707
b	0	0	3	2	-1	3	3	3
R (μm)	17.56	20.66	18.60	28.93	22.73	18.60	24.32	21.80
T (ms)	42.5	70	127	212.9	230	266	670	710
Q_{in} (μL h^{-1})	4.27	4.79	4.76	4.82	4.96	4.23	4.77	4.87

Table S2. Data for calculating the inner flow rate Q_{in} measured from the video. In the row of "Type", "De", "Con-de" and "In-con-de" represent droplet sequence with size distribution in the form of "Descending", "Constant-decreasing" and "Increasing-constant-decreasing", respectively. For L = 439.4 μm, b = -1 means that the left sequence with apex angle $2\theta_1$ and the right sequence with $2\theta_2$ share the same biggest droplet. In this case, the droplet firstly increases in size, and then directly decreases without keeping constant.
